# Src activation by cGMP/PKG II in osteoblasts: characterization of a mechano-sensitive signalling complex

**DOI:** 10.1186/1471-2210-11-S1-O24

**Published:** 2011-08-01

**Authors:** Hema Rangaswami, Raphaela Schwappacher, Nisha Marathe, Darren E Casteel, Bodo Haas, Alexander Pfeifer, Sanford Shattil, Gerry Boss, Renate B Pilz

**Affiliations:** 1 Department of Medicine, University of California at San Diego, La Jolla, CA 92093, USA; 2 Institute for Pharmacology and Biomedical Center, University of Bonn, 53105 Bonn, Germany

## Background

Mechanical stimulation of bone cells is critical for maintaining bone mass and strength, and a better understanding of how mechanical stimuli are converted into intracellular signals to activate an anabolic program in osteoblasts/cytes is fundamental to improving treatments for osteo-degenerative diseases [1,2]. Weight bearing and locomotion stimulate interstitial fluid flow through the bone canalicular system, and the resultant shear stress is thought to be a major mechanism whereby mechanical forces stimulate osteoblast/osteocyte growth and differentiation [1,2]. In primary osteoblasts and osteoblast/cyte-like cell lines, fluid shear stress induces rapid expression of *c-fos*, *fra-1*, *fra-2*, and *fosB/ΔfosB* mRNAs [3]; these genes encode transcriptional regulators important for osteoblast proliferation and differentiation, as demonstrated by the phenotypes of mice that over-express or lack these proteins, respectively [4]. We have previously shown that fluid shear stress increases osteoblast/cyte nitric oxide (NO) production, leading to increased cGMP synthesis and activation of cGMP-dependent protein kinases (PKGs). The NO/cGMP/PKG signaling pathway is required for shear-induced expression of all four *fos* family genes, and induction of these genes is mediated through activation of the mitogen-activated protein kinases Erk1/2 [3]. However, molecular mechanisms leading to Erk activation in shear-stressed osteoblasts are largely unknown.

## Results

We have now defined the events leading from shear stress activation of NO/cGMP/PKG II to the activation of Src [5]; we show that this pathway is required for Erk activation, and controls osteoblast proliferation and survival. We found a novel link between NO/cGMP/PKG and β3 integrins, the key mechano-sensors in bone, and show that PKG II activates β3-associated Src by activating the tyrosine phosphatase Shp-1. PKG II directly phosphorylates and stimulates Shp-1 activity, which de-phosphorylates a C-terminal, inhibitory phosphorylation site on Src, leading to Src activation. Fluid shear stress triggers PKG II, Src, and Shp-1/2 recruitment to a mechano-sensitive complex containing β3 integrins, defining a novel “mechanosome”. We found that PKG II-null mice have defective osteoblast Src/Erk signaling, decreased Erk-dependent gene expression in bone, and impaired osteoblast-dependent, membranous bone formation. These results complement previous studies in PKG II-deficient mice, which showed defective chondroblast differentiation and endochondral bone formation, and studies in NO synthase-deficient mice, which demonstrated an important role of NO in osteoblast biology [6,7].

## Conclusion

Our findings reveal crosstalk between NO/cGMP/PKG and integrins, establish a new mechanism of Src activation, and fill a gap in our understanding of how mechanical forces acting on cell-matrix adhesions are translated into cellular responses. Since Src controls Erk, a key regulator of osteoblast growth and survival, our results support using PKG-activating drugs as mechano-mimetics for treating osteoporosis.
